# Aberrant expression of S-SCAM causes the loss of GABAergic synapses in hippocampal neurons

**DOI:** 10.1038/s41598-019-57053-y

**Published:** 2020-01-09

**Authors:** Seung Min Shin, Samantha Skaar, Eric Danielson, Sang H. Lee

**Affiliations:** 1 Department of Pharmacology and Toxicology, Medical College of Wisconsin, 8701 Watertown Plank Road, Milwaukee, Wisconsin USA; 2 Neuroscience Research Center, Medical College of Wisconsin, 8701 Watertown Plank Road, Milwaukee, Wisconsin USA

**Keywords:** Cellular neuroscience, Synaptic plasticity

## Abstract

The duplication and deletion mutations of the *S-SCAM/MAGI-2* gene are associated with schizophrenia and infantile spasms, respectively. S-SCAM is a unique synaptic scaffolding protein that localizes to both excitatory and GABAergic synapses. However, consequences of aberrant *S-SCAM* expression on GABAergic synapses is little studied. Here we report the effect of S-SCAM knockdown and overexpression on GABAergic synapses. S-SCAM knockdown in cultured hippocampal neurons caused a drastic loss of both pre- and post-synaptic components of GABAergic synapses, indicating its essential role in GABAergic synapse formation and maintenance. Surprisingly, S-SCAM overexpression also attenuated GABAergic synapses, but the effect is mediated by the loss of postsynaptic GABA_A_ receptors, gephyrin, and neuroligin 2 and does not involve presynaptic component vesicular GABA transporters. Overexpression studies using S-SCAM mutants with various domain deletions indicated that GABAergic synapse loss correlates with their ability to increase excitatory synaptic function. Consistently, AMPA receptor antagonist CNQX or calcineurin inhibitor FK506 abolished the S-SCAM overexpression-induced loss of GABA_A_ receptors, supporting that GABAergic synapse loss by S-SCAM overexpression is due to the activity-induced dispersal of synaptic GABA_A_ receptors. These results suggest that abnormal S-SCAM protein levels disrupt excitation/inhibition balance in neurons, which may explain the pathogenic nature of *S-SCAM* copy number variations.

## Introduction

Maintaining proper balance in excitation and inhibition (E/I) is critical for circuit functionality and thus brain function^1^. Disruption of the E/I balance is implicated for numerous diseases including schizophrenia^2^, autism^3^, and epilepsy^4^. However, not much is known for the cell-autonomous mechanism by which the balance of excitatory and inhibitory synapses is maintained in neurons^5–7^.

Scaffolding proteins play crucial roles at synapses in the assembly of signaling complexes, trafficking and clustering of receptors, stabilization of synapses, and dynamic turnover of synaptic components^8^. Synaptic scaffolding molecule (S-SCAM), also known as membrane-associated guanylate kinase inverted 2 (MAGI-2), is a major synaptic scaffolding protein that consists of six PDZ domains, one guanylate kinase (GK) domain, and two WW domains^9^. S-SCAM interacts with numerous proteins at synapses including transmembrane AMPA receptor regulatory protein (TARP), guanylate kinase associated protein (GKAP), neuroligins, Axin, β-catenin, and ErbB4^10^. Overexpression of S-SCAM in excitatory neurons specifically enhances AMPA receptor (AMPAR)-mediated synaptic transmission through TARP^11,12^, without altering NMDA receptor (NMDAR) and presynaptic function^11^. Conversely, S-SCAM knockdown induced the loss of synaptic AMPARs and excitatory synapses. Therefore, S-SCAM is a critical scaffolding protein that controls the strength of excitatory synaptic transmission by regulating the amount of AMPARs at synapses. Importantly, the overexpression of S-SCAM not only enhanced excitatory synaptic transmission but also impaired synaptic plasticity^11,13^.

Remarkably, elevated S-SCAM levels in the forebrains, simulating *S-SCAM* duplication conditions in schizophrenia^14^, led to the manifestation of a remarkably wide array of schizophrenia -related behavioral endophenotypes modeling all three domains of schizophrenia symptoms in S-SCAM transgenic mice^13^. In addition to the behavioral endophenotypes, S-SCAM transgenic mice also feature morphological alterations found in schizophrenia, including a reduced number of dendritic spines and enlarged lateral ventricles^13^.

S-SCAM also localizes to GABAergic synapses, interacts with key postsynaptic components such as β-dystroglycan, IgSF9b, and neuroligin 2 (NL2)^15,16^, and is implicated for the assembly of inhibitory synapses in interneurons^16^. However, its role in GABAergic synapses in pyramidal neurons has not been studied yet. Interestingly, haplodeficiency of the *S-SCAM* gene is associated with infantile spasms^17^, the most common and severe form of epilepsy in infants and childhood, suggesting the potential pathogenic role of S-SCAM deficiency in GABAergic function^18^. Moreover, S-SCAM transgenic mice showed reduced GABA_A_ receptor α1 levels, specifically in the synaptosomal fraction (biochemical correlates of synapses) without alterations in its total protein levels^13^, indicating the possibility of defects in GABAergic synapses.

In this paper, we describe the effect of altering S-SCAM protein levels, mimicking the conditions in schizophrenia or infantile spasms, in cultured rat hippocampal neurons on the GABAergic synapses using immunocytochemistry combined with molecular genetic, pharmacological, and biochemical approaches. Our studies reveal the profound importance of S-SCAM in maintaining the proper balance of excitatory and inhibitory synapses in neurons and provide a clue to the pathogenic properties of *S-SCAM* copy number variations.

## Results

### S-SCAM knockdown causes the loss of GABAergic synapses in cultured hippocampal neurons

**Figure 1:**
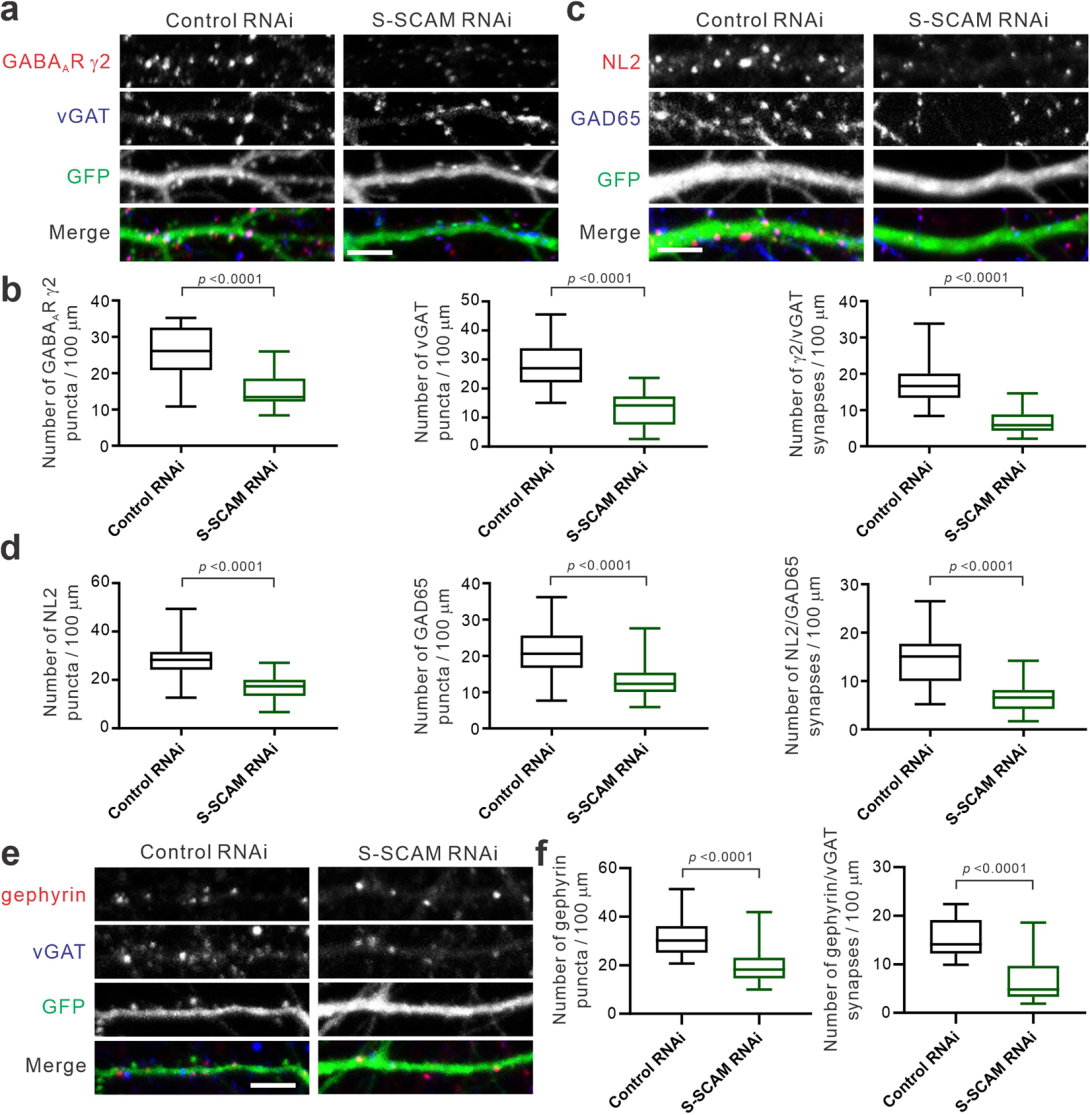
S-SCAM Knockdown reduces the number of GABAergic synapses. Cultured rat hippocampal neurons (div 14) were transfected with plasmids expressing either control shRNA or S-SCAM-specific shRNA (RNAi). After 3 days post-transfection, neurons were fixed and stained for indicated GABA synapse markers. (**a**) Representative immunofluorescent images of GABA_A_R γ2 and vGAT in the dendrites of hippocampal neurons transfected with control or S-SCAM shRNAs. (**b**) Quantification of puncta densities of GABA_A_R γ2 (*left*), vGAT (*middle*), and co-localized GABA_A_R γ2 and vGAT (*right*). *n* = 24 (control) and 23 (S-SCAM RNAi) from triplicate experiments. Unpaired t test. *F* values are: for GABA_A_R γ2, *F*_(1,45)_ = 2.128, *p* = 0.0813; for vGAT, *F*_(1,44)_ = 1.543, *p* = 0.3161; for γ2/vGAT, *F*_(1,34.9)_ = 2.653, *p* = 0.0307. (**c**) Representative immunofluorescent images of NL2 and GAD65 in the dendrites of hippocampal neurons transfected with control or S-SCAM RNAi. (**d**) Quantification of puncta densities of NL2 (*left*), GAD65 (*middle*), and co-localized NL2 and GAD65 (*right*). *n* = 35 (control) and 33 (S-SCAM RNAi). NL2, *F*_(1,60.31)_ = 2.140, *p* = 0.0331; GAD65, *F*_(1,66)_ = 1.932, *p* = 0.0643; NL2/GAD65, *F*_(1,52.48)_ = 3.527, *p* = 0.0005. (**e**) Representative immunofluorescent images of gephyrin and vGAT. (**f**) Quantification of puncta densities of gephyrin (*left*) and co-localized gephyrin and vGAT (*right*). *n* = 29 (control) and 27 (S-SCAM RNAi). gephyrin, *F*_(1,47)_ = 1.615, *p* = 0.2545; gephyrin/vGAT, *F*_(1,54)_ = 1.572, *p* = 0.2436. Scale bars, 5 μm.

To study the role of S-SCAM in GABAergic synapses in pyramidal neurons, we used the shRNA-mediated S-SCAM knockdown (RNAi) approach that successfully identified the role of S-SCAM in glutamatergic synapses^11^. The specificity and efficacy of the S-SCAM shRNAs were demonstrated previously^11^. First, we performed immunocytochemistry of cultured rat hippocampal neurons to examine postsynaptic GABA_A_ receptor γ2 (GABA_A_R γ2; the most common subunit of GABA_A_Rs) and presynaptic vesicular GABA transporter (vGAT), which are markers for GABAergic synapses. As shown in Fig. 1a,b, S-SCAM RNAi greatly reduced the numbers of both GABA_A_R γ2 and vGAT puncta in the dendrites (57% and 43% compared to control, respectively). Moreover, S-SCAM RNAi also reduced the number of co-localized GABA_A_R γ2 and vGAT puncta that represent GABAergic synapses (17.3 ± 0.9 vs 6.6 ± 0.7 per 100 μm; 38% of control). To corroborate the findings, we also examined NL2 and glutamate decarboxylase 65 (GAD65) as additional markers of GABAergic synapses. As shown in Fig. 1c,d, S-SCAM RNAi significantly reduced the puncta numbers of NL2 and GAD65 (60% and 62% compared to control, respectively) and decreased the densities of colocalized NL2/GAD65 puncta (14.2 ± 0.9 vs 6.5 ± 0.5 per 100 μm; 46% of control). S-SCAM RNAi also decreased gephyrin puncta density (61% of control) and the densities of colocalized gephyrin/vGAT puncta (15.5 ± 0.7 vs 6.6 ± 1.0 per 100 μm; 43% of control) (Fig. 1e,f). These results indicate that the loss of S-SCAM greatly reduces the number of GABAergic synapses and thus suggest that S-SCAM is also required for the formation and/or maintenance of GABAergic synapses as well as glutamatergic synapses.

### S-SCAM overexpression reduces GABAergic synapse numbers in hippocampal neurons

**Figure 2:**
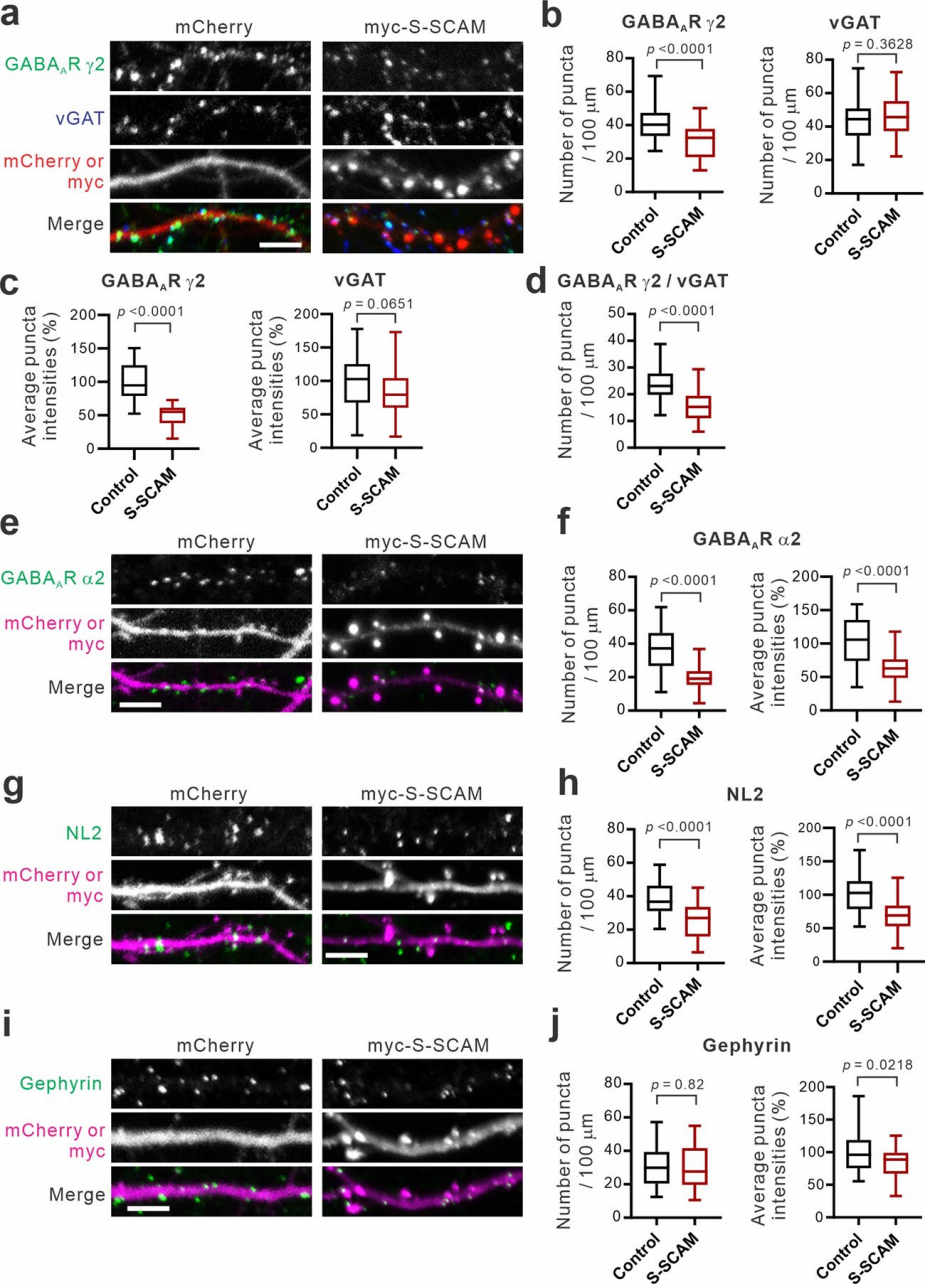
S-SCAM overexpression causes the loss of GABAergic synapses. (**a**) Representative immunofluorescent images of GABA_A_R γ2 and vGAT in the dendrites of hippocampal neurons transfected with plasmids expressing either control (mCherry) or myc-S-SCAM. (**b−d**) Quantification of densities (**b**) and intensities (**c**) of GABA_A_R γ2 and vGAT puncta, and co-localized GABA_A_R γ2 and vGAT puncta densities (**d**). *n* = 23 (control) and 25 (S-SCAM) from triplicate experiments. Unpaired t test. *F* values are: GABA_A_R γ2, *F*_(1,46)_ = 1.053, *p* = 0.8985; vGAT, *F*_(1,90)_ = 1.035, *p* = 0.9135 (**b**), GABA_A_R γ2, *F*_(1,30.17)_ = 3.786, *p* = 0.0019; vGAT, *F*_(1,71)_ = 1.044, *p* = 0.8936 (**c**), GABA_A_R γ2/vGAT, *F*_(1,54)_ = 1.335, *p* = 0.4577 (**d**). (**e–j**) Representative immunofluorescent images of GABA_A_R α2 (**e**), NL2 (**g**), and gephyrin (**i**) in the dendrites of hippocampal neurons transfected with mCherry or myc-S-SCAM and quantification of puncta densities and average intensities of GABA_A_R α2 (**f**), NL2 (**h**), and gephyrin (**j**). *n* = 23–26 per condition. Unpaired t test. *F* values are: GABA_A_R α2 density, *F*_(1,44)_ = 2.162, *p* = 0.0773; intensity, *F*_(1,76)_ = 1.694, *p* = 0.1050 (**f**); NL2 density, *F*_(1,47)_ = 1.069, *p* = 0.8801; intensity, *F*_(1,70)_ = 1.542, *p* = 0.2035 (**h**); gephyrin density, *F*_(1,49)_ = 1.005, *p* = 0.9915, intensity, *F*_(1,76)_ = 1.544, *p* = 0.1852 (**j**). Scale bars, 5 μm.

We next investigated the effect of S-SCAM overexpression on inhibitory synapses in cultured rat hippocampal neurons. Several postsynaptic markers such as GABA_A_R γ2, GABA_A_R α2, NL2, gephyrin, and presynaptic vGAT were examined. Unlike excitatory synapses, to our surprise, S-SCAM overexpression significantly reduced both puncta densities and average intensities of GABA_A_R γ2 (74% and 50% of control, respectively; Fig. 2a–c), GABA_A_R α2 (55% and 62% of control, respectively; Fig. 2e,f), and NL2 (63% and 69% of control, respectively; Fig. 2g,h). On the other hand, S-SCAM overexpression did not significantly alter the densities and average intensities of presynaptic vGAT puncta (Fig. 2a–c). GABAergic synapse numbers determined by counting colocalized GABA_A_R γ2 and vGAT puncta densities also showed significant reduction (64% of control; Fig. 2d). Interestingly, S-SCAM overexpression did not affect gephyrin puncta densities but significantly reduced average intensities of gephyrin clusters (Fig. 2i,j). These results indicate that S-SCAM overexpression causes the reduction in the number of “immunocytochemical” GABAergic synapses, which is mainly caused by postsynaptic and cell-autonomous effect.

### Generation of S-SCAM deletion mutants and their dendritic spine targeting

**Figure 3:**
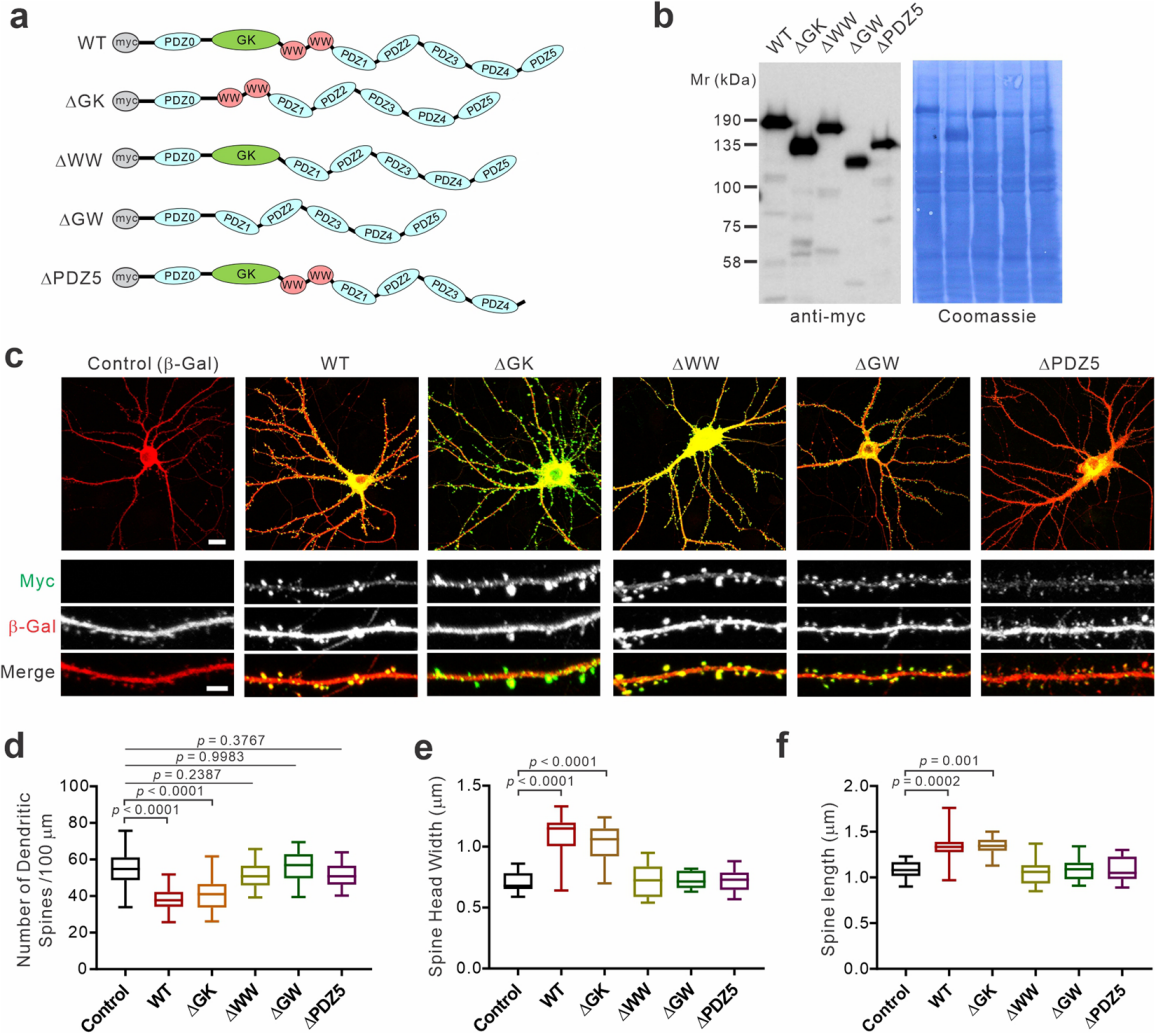
Excitatory synaptic targeting of S-SCAM deletion mutants and their effect on dendritic spines. (**a**) Schematic diagrams of S-SCAM deletion mutants. (**b**) Full-length expression of various S-SCAM deletion mutants expressed in COS-7 cells. *Left* panel, immunoblot probed with anti-myc antibody; *right* panel, Coomassie Blue stained blot. (**c**) Dendritic spine targeting of S-SCAM deletion mutants. Scale bars, 10 μm (low magnification images) or 5 μm (dendrites). (**d**) Quantified effect of S-SCAM deletion mutant overexpression on dendritic spine density. *n* = 42 (Control), 46 (WT), 41 (ΔGK), 41 (ΔWW), 39 (ΔGW), 45 (ΔPDZ5). One-way ANOVA, *F*_(5,248)_ = 41.86, *p* < 0.0001, Tukey’s post-hoc test. (**e,f**) Quantified effect of S-SCAM deletion mutant overexpression on dendritic spine width (**e**) and length (**f**). One-way ANOVA, *F*_(5,65)_ = 25.59, *p* < 0.0001 (**e**), *F*_(5,60)_ = 11.94, *p* < 0.0001 (**f**), Tukey’s post-hoc test.

Increased glutamatergic activity could induce hetero-synaptic plasticity of reducing GABAergic transmission^19–23^. Since S-SCAM overexpression increases glutamatergic activity^11,13^, we next thought the possibility that hyper-glutamatergic activity is responsible for the loss of GABAergic synapses in S-SCAM overexpressing neurons. To investigate this, first, we determined specific domains of S-SCAM required for the enhancement of glutamatergic activity. Four different deletion mutants, ΔGK (mutant with a deletion in the guanylate kinase domain), ΔWW (mutant with a deletion in the two WW domains), ΔGW (mutant with a deletion in both GK and WW domains), and ΔPDZ5 (mutant with a deletion in the 5^th^ PDZ domain) were created (Fig. 3a). Full-length protein expression of the four mutants was confirmed in COS-7 cells (Fig. 3b). When these mutants were transfected into hippocampal neurons together with a cell-fill marker β-galactosidase (β-Gal), they all showed comparable expression levels to WT except for ΔPDZ5 that showed relatively weaker expression (Fig. 3c). Importantly, all mutants showed similar staining patterns with much higher fluorescent intensities in dendritic spines than in dendritic shaft, indicating their dendritic spine targeting (Fig. 3c). A minor portion of these puncta was found in dendritic shafts, usually smaller in size than the ones localized to spines. Since most inhibitory synapses are formed on the shaft of dendrites^24^, they represent S-SCAM populations localized to GABAergic synapses. These results are consistent with previous reports showing that ~35% of S-SCAM puncta are detected at inhibitory synapses^15,16^.

### WW and PDZ5 domains of S-SCAM are required to increase dendritic spine sizes

Having confirmed the spine targeting of these mutants, we next analyzed the effect of S-SCAM mutants on the density and size of dendritic spines. Dendritic spine size is directly correlated with synaptic strength^25^. Consistent with our previous report^11^, S-SCAM WT overexpression reduced the density of dendritic spines by ~30% (Fig. 3d), which is likely due to the reduction of new spine formation, rather than removing the existing spines. Overexpression of ΔGK mutant showed similar reduction in dendritic spine densities to WT. However, ΔWW, ΔGW, and ΔPDZ5 mutants did not show such effect on dendritic spine density. Essentially identical results were obtained for the effect of these mutants on dendritic spine head width and length (Fig. 3e,f). While WT and ΔGK significantly increased the dendritic spine width (1.56- and 1.44-fold, respectively) and length (both 1.23-fold), ΔWW, ΔGW, and ΔPDZ5 mutants failed to produce such effect. Therefore, WW and PDZ5 domains of S-SCAM are required for the increase of dendritic spine size, but GK domain is not.

### Effect of S-SCAM deletion mutants on surface AMPAR levels

**Figure 4:**
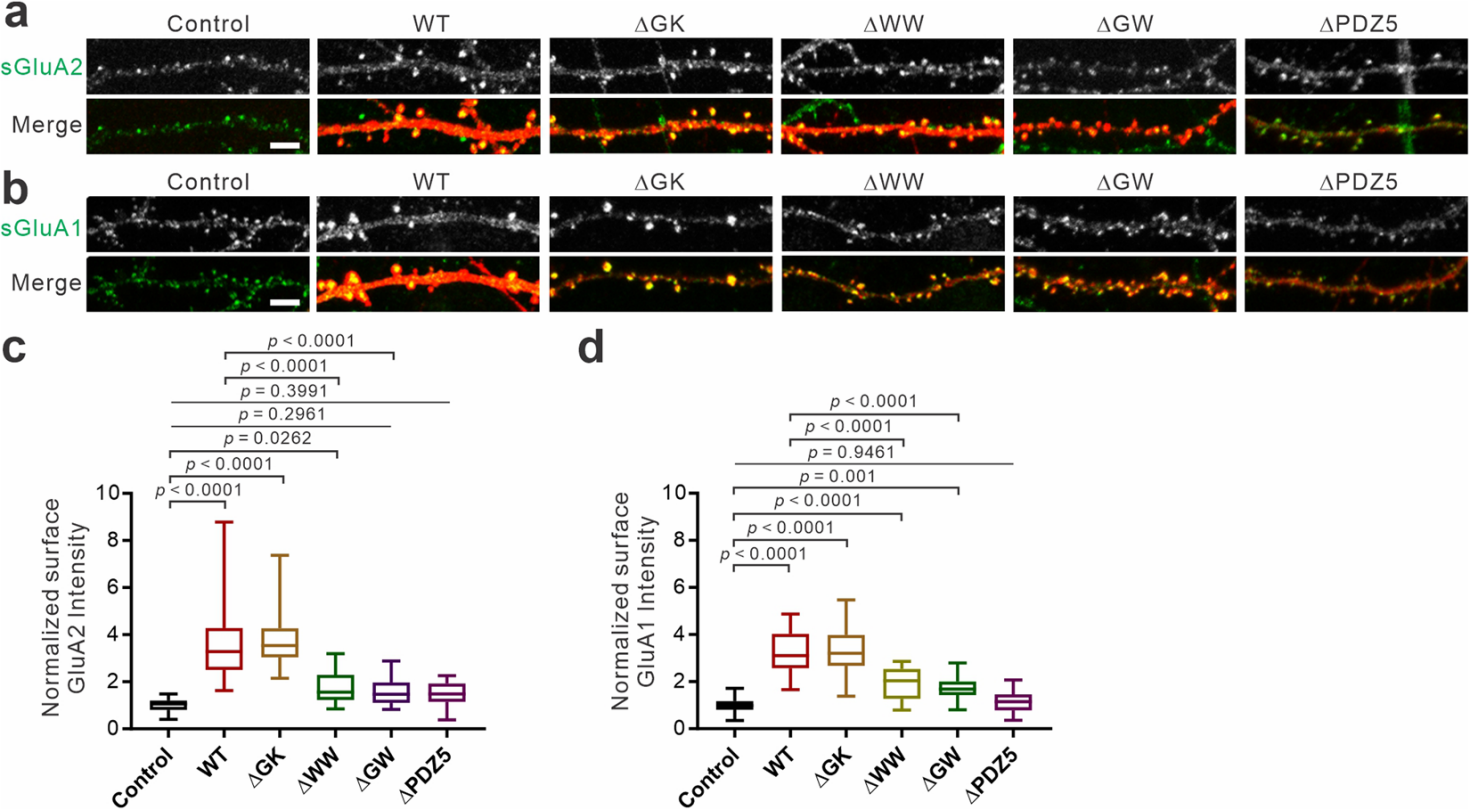
Effect of S-SCAM deletion mutants on surface AMPA receptor levels. (**a**,**b**) Representative images of hippocampal neurons transfected with various S-SCAM deletion mutants stained for sGluA2 (**a**) and sGluA1(**b**). (**c,d**) Quantification of S-SCAM deletion mutant overexpression effect on the average intensities of sGluA2 (**c**) and sGluA1 (**d**). *n* = 27 and 47 (Control), 27 and 27 (WT), 28 and 23 (ΔGK), 27 and 20 (ΔWW), 23 and 29 (ΔGW), 24 and 20 (ΔPDZ5) for sGluA2 and sGluA1, respectively. One-way ANOVA, *F*_(5,150)_ = 43.63, *p* < 0.0001 (**c**), *F*_(5,160)_ = 71.15, *p* < 0.0001 (**d**), Tukey’s post-hoc test. Scale bars, 5 μm.

We next examined the effect of S-SCAM deletion mutant overexpression on the surface AMPAR levels. As shown in Fig. 4a,b, surface GluA2 (sGluA2) and sGluA1, two major AMPAR subunits expressed in the hippocampus, are highly concentrated in dendritic spines. Consistent with the dendritic spine data (Fig. 3), when compared to control non-transfected neighboring neurons, WT and ΔGK-overexpressing neurons showed greater than 3-fold increases in sGluA2 and sGluA1 staining intensities (Fig. 4a–c). However, neurons transfected with ΔWW, ΔGW, and ΔPDZ5 did not show such increase of sGluA2 (Fig. 4a,c). Notably, unlike sGluA2, ΔWW and ΔGW mutants increased sGluA1 staining compared to the control (190 ± 16% and 167 ± 7%, respectively), but these increases were significantly smaller compared to WT and ΔGK (323 ± 17% and 325 ± 21%, respectively; Fig. 4b,d). ΔPDZ5 did not significantly affect both sGluA2 and sGluA1 levels. All in all, these results indicate that ΔWW and ΔPDZ5 are S-SCAM mutants having a defect in the ability to enhance glutamatergic function.

### Effect of S-SCAM deletion mutants on GABAergic synapses

**Figure 5:**
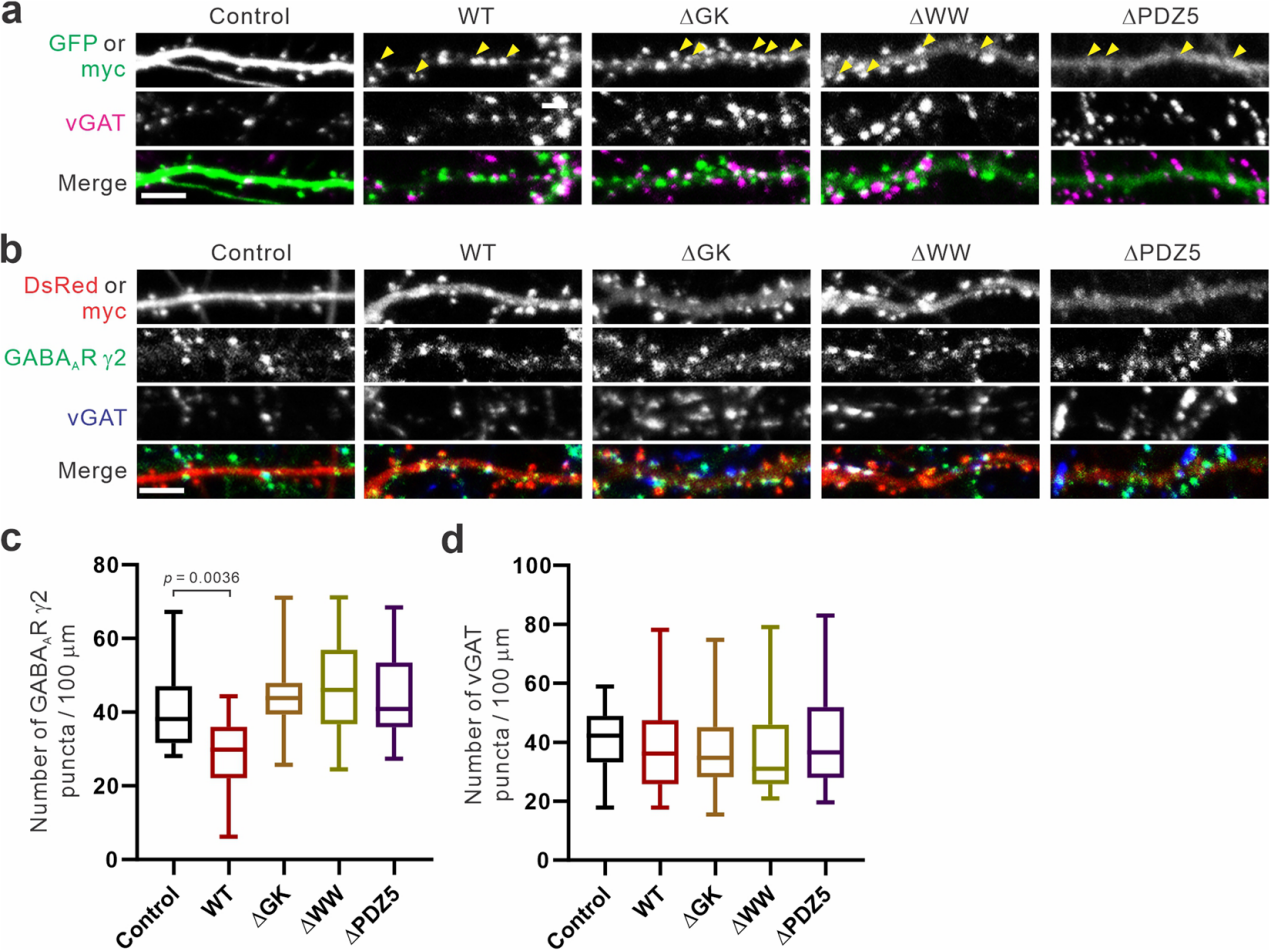
Effect of S-SCAM deletion mutants on GABA_A_ receptor clusters. (**a,b**) Representative immunocytochemical images of hippocampal neurons transfected with various S-SCAM deletion showing vGAT (**a**) and vGAT and GABA_A_R γ2 (**b**). Yellow arrowheads in (**a**) mark co-localized myc-S-SCAM and vGAT. (**c,d**) Quantification of S-SCAM deletion mutant overexpression effect on the densities of GABA_A_R γ2 (**c**) and vGAT puncta (**d**). *n* = 27 (Control), 25 (WT), 26 (ΔGK), 21 (ΔWW), 20 (ΔPDZ5). One-way ANOVA, *F*_(4,114)_ = 9.787, *p* < 0.0001 (**c**), *F*_(4,152)_ = 0.4408, *p* = 0.779 (**d**), Tukey’s post-hoc test. Scale bars, 5 μm.

Having obtained S-SCAM mutants unable to enhance glutamatergic synaptic function, we compared these mutants for their ability to attenuate GABAergic synapses. All mutants showed localization to GABAergic synapses indistinguishable from WT, as examined by their co-localization with vGAT (Fig. 5a; marked by arrowheads). In contrast to WT, the overexpression of S-SCAM deletion mutants, ΔWW and ΔPDZ5, did not reduce the density of GABA_A_R γ2 puncta. Interestingly, the ΔGK mutant also did not affect the density of GABA_A_R γ2 puncta, despite increasing dendritic spine sizes and sGluA intensities (Figs. 3e,f and 4). Consistent with WT data (Fig. 2), none of these mutants’ overexpression affected the densities of presynaptic vGAT puncta (Fig. 5d).

### Excitatory activity mediated by AMPARs is required for the S-SCAM-induced loss of GABA_A_R γ2 clusters

**Figure 6:**
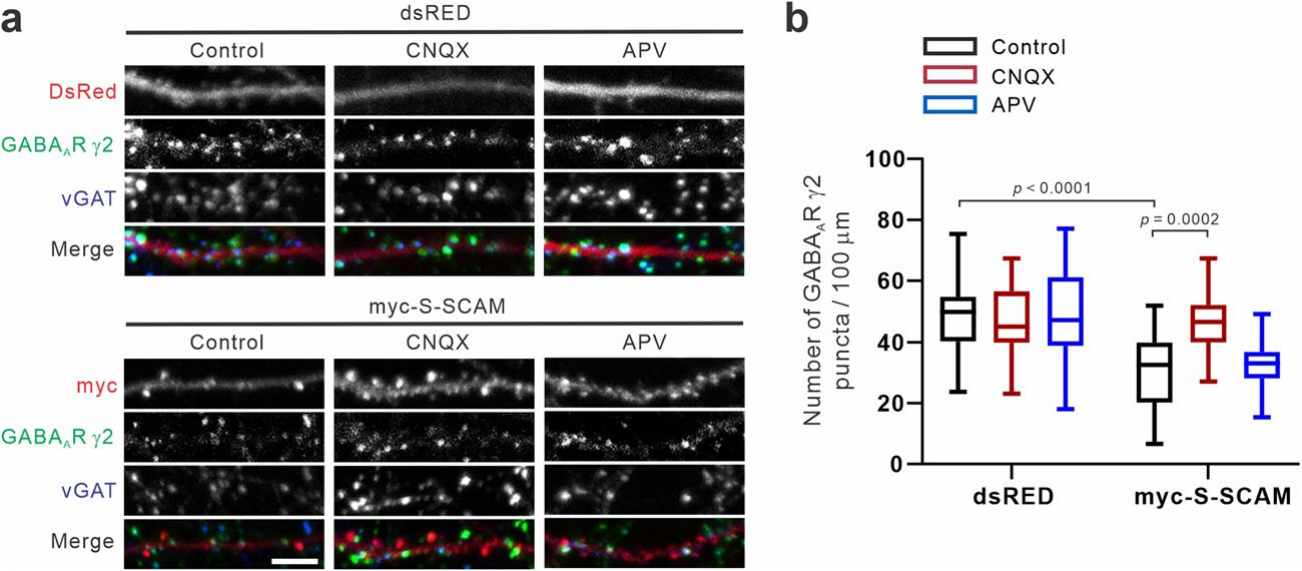
Effect of glutamate antagonists, CNQX or APV, on S-SCAM overexpression-induced loss of GABA_A_ receptor clusters. (**a**) Representative GABA_A_R γ2 and vGAT immunocytochemical images of hippocampal neurons transfected with DsRed or myc-S-SCAM treated with either DMSO (Control), APV (100 μM), or CNQX (50 μM). (**b**) Quantification of the glutamate antagonist effects on GABA_A_R γ2 clusters. *n* = 26 (DsRed, Control), 23 (DsRed, CNQX), 24 (DsRed, APV), 30 (S-SCAM, Control), 22 (S-SCAM, APV), 30 (S-SCAM, CNQX). Two-way ANOVA, interaction *F*_(2,149)_ = 6.989, *p* = 0.0013, treatment group *F*_(1,149)_ = 32.85, *p* < 0.0001, construct group *F*_(2,149)_ = 4.959, *p* = 0.0082, Tukey’s post-hoc test. Scale bars, 5 μm.

To further demonstrate the role of glutamatergic activity in the S-SCAM overexpression-induced loss of GABAergic synapses, we next examined the effect of glutamate receptor antagonists that block excitatory activity. In control neurons transfected with DsRed, the addition of neither AMPAR antagonist (6-cyano-7-nitroquinoxaline-2,3-dione; CNQX) nor NMDAR antagonist (D-2-amino-5-phosphonovaleric acid; APV) affected the density of GABA_A_R γ2 puncta significantly (Fig. 6a *upper panel* and Fig. 6b). In contrast, CNQX completely blocked the reduction of GABA_A_R γ2 puncta densities in myc-S-SCAM-transfected neurons. However, APV did not affect the reduction of GABA_A_R γ2 puncta densities in myc-S-SCAM-transfected neurons (Fig. 6a *bottom panel* and Fig. 6b). Therefore, these data suggest that AMPAR-mediated excitatory activity is required for the S-SCAM overexpression-induced reduction of GABAergic synapses.

### Calcineurin mediates the S-SCAM overexpression-induced loss of GABA_A_R γ2 clusters

**Figure 7:**
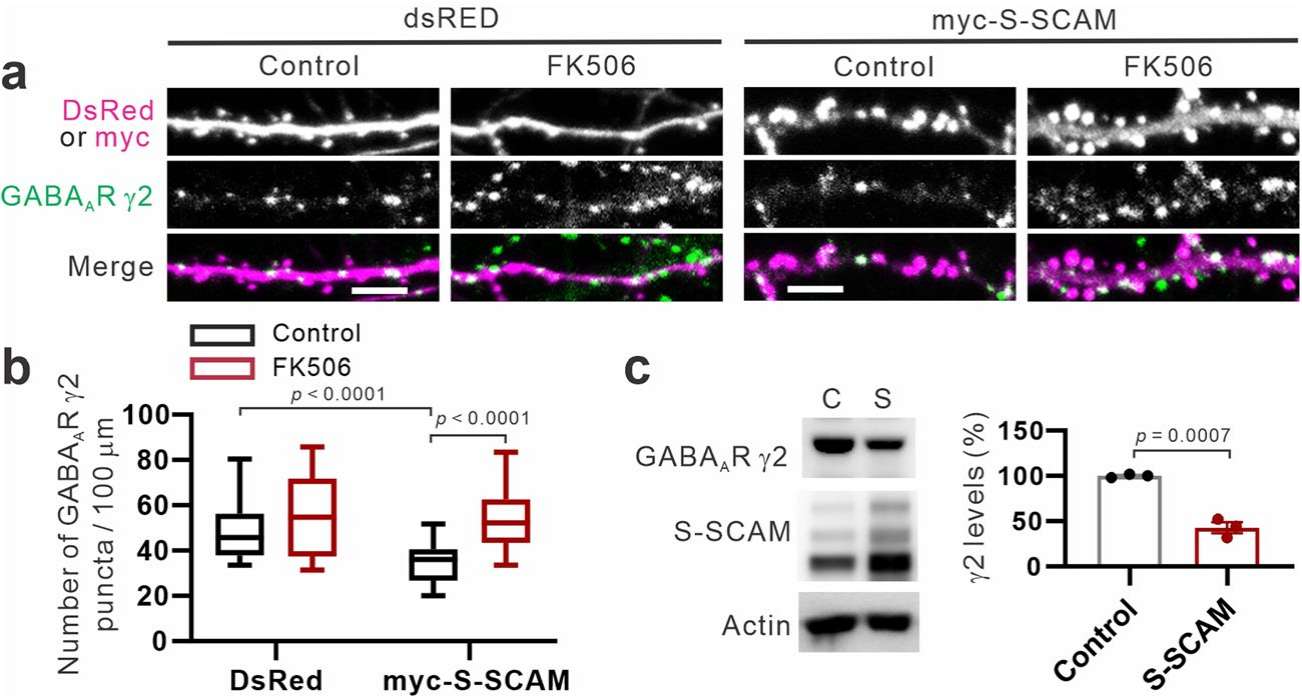
Effect of calcineurin inhibitor FK506 on the S-SCAM overexpression-induced loss of GABA_A_ γ2 receptor clusters. (**a**) Representative images of GABA_A_R γ2 in hippocampal neurons transfected with DsRed or myc-S-SCAM treated with either DMSO (Control) or FK506 (50 nM). (**b**) Quantification of the FK506 effect on GABA_A_R γ2 clusters. *n* = 27 (DsRed Control), 26 (DsRed FK506), 25 (S-SCAM Control), 22 (S-SCAM FK506). Two-way ANOVA, interaction *F*_(1,96)_ = 6.217, *p* = 0.0144, treatment group *F*_(1,96)_ = 5.383, *p* = 0.0224, construct group *F*_(1,96)_ = 21.69, *p* < 0.0001, Tukey’s post-hoc test. (**c**) Effect of SCAM overexpression on the GABA_A_R γ2 protein levels. Actin is used as a loading control. *n* = 3, Unpaired t test. *F*_(1,4)_ = 26.63, *p* = 0.072. Scale bars, 5 μm.

Lastly, we examined the involvement of calcineurin that is a critical mediator of the activity-dependent removal of synaptic GABA_A_Rs^19,20,22,26,27^. To achieve this, we used a well-established specific inhibitor of calcineurin, FK506. The addition of FK506 did not affect the number of GABA_A_R γ2 puncta in control DsRed-transfected neurons (Fig. 7a *upper panel* and Fig. 7b). However, FK506 completely blocked the loss of GABA_A_R γ2 puncta in S-SCAM-overexpressing neurons (Fig. 7a *upper panel* and Fig. 7b), indicating that calcineurin activity is required for the GABAergic synapse loss. To gain an insight on the fate of GABA_A_Rs removed from synapses, we next examined the effect of S-SCAM overexpression on the protein levels of GABA_A_R γ2 using Sindbis viruses expressing GFP-S-SCAM^11,28^. In neurons overexpressing S-SCAM, the amount of total GABA_A_R γ2 is greatly reduced compared to control neurons (42.8 ± 5.9%; Fig. 7c; see also Supplementary Information). These results suggest that S-SCAM overexpression causes the reduction of GABAergic synapses by promoting calcineurin-dependent dispersal and subsequent degradation of GABA_A_Rs^23,29^.

## Discussion

There are two major findings from these studies: First, S-SCAM is required for the proper formation and/or maintenance of GABAergic synapses in pyramidal cells. Second, abnormally high levels of S-SCAM lead to the weakening of GABAergic synapses. These findings not only illustrate the functional importance of postsynaptic scaffolding protein S-SCAM in the proper maintenance of GABAergic synapses in principal neurons but also have implications for the pathogenesis mechanisms associated with schizophrenia and infantile spasms.

The reduced levels of S-SCAM induced by S-SCAM knockdown led to the loss of not only postsynaptic components but also presynaptic components of GABAergic synapses, suggesting the complete loss of GABA synapses. In the case of interneurons, it was reported that the scaffolding function of S-SCAM by bridging together two adhesion molecules, IgSF9b and NL2, is essential for the proper formation of GABAergic synapses^16^. Similarly, our results indicate that proper postsynaptic clustering of NL2 requires S-SCAM in pyramidal neurons. NL2 is not only required for the formation of GABAergic synapses but is also sufficient for inducing presynaptic differentiation^30,31^. Considering the essential and crucial role of NL2 in the formation of GABAergic synapses, therefore, NL2 loss seems to be the main cause for the loss of inhibitory synapses in neurons with reduced levels of S-SCAM.

We have previously shown that S-SCAM overexpression leads to the increased accumulation of AMPARs at synapses and greatly enhances glutamatergic synaptic transmission by nearly 2-fold^11,13^. In sharp contrast, S-SCAM overexpression leads to the loss of postsynaptic components of GABAergic synapses such as GABA_A_Rs, NL2, and gephyrin. However, S-SCAM overexpression does not affect presynaptic side of GABAergic synapses as determined by presynaptic vGAT, suggesting cell-autonomous effects. Therefore, S-SCAM overexpression triggers a profound weakening and functional loss of inhibitory synapses but may not cause a bona fide elimination. Importantly, activity-dependent turnover of inhibitory synapses as monitored by gephyrin is a reversible process – disappearing and reappearing frequently at the same sites^32^. Therefore, one interesting possibility is that activity-dependent dynamics of inhibitory synapses may involve the turnover of mostly postsynaptic components. In line with this notion, chronic hyperpolarization of an individual postsynaptic neuron does not change presynaptic GAD density and intensity^33^. It would require further extensive studies employing robust approaches including electrophysiology, real-time imaging of both pre- and post-synaptic components, and electron microscopic analyses of synapses to fully characterize the processes.

What is the mechanism by which S-SCAM overexpression induces the attenuation of GABAergic synapses? Hyper-glutamatergic activity induced-loss of GABAergic synapses is initiated by the lateral diffusion of GABA_A_Rs out of synapses, which requires calcineurin activity^19^. GABA_A_Rs moved to extra-synaptic area of the plasma membrane undergo internalization and are degraded in lysosomes^23,34^. Consistent with these findings, S-SCAM overexpression causes a calcineurin-dependent loss and drastic reduction of total protein levels of GABA_A_R γ2, which suggests its degradation. Therefore, hyper-glutamatergic activity is likely the main cause of GABAergic synapse attenuation in S-SCAM-overexpressing neurons. Notably, S-SCAM overexpression-induced loss of GABA_A_Rs requires AMPAR activity but not NMDAR activity, which is apparently different from previous studies suggesting the key role of NMDARs in the activity-induced dispersal of GABA_A_Rs^19–21^. However, these studies were performed by examining the effect of exogenous NMDA addition and did not examine the contribution of AMPARs. Activation of AMPARs could also lead to the increase in intracellular calcium via L-type voltage-gated calcium channels, which is directly coupled to AKAP-anchored calcineurin^35,36^. Therefore, our studies uncover a novel signaling pathway for GABAergic synapse modulation. The exact molecular mechanism by which dephosphorylation of GABA_A_R γ2, an essential subunit of GABA_A_Rs for postsynaptic clustering^37^, causes the dispersal is unknown. Interestingly, the GK domain of S-SCAM, although not necessary for the enhancement of glutamatergic synapses, is required for the attenuation of GABAergic synapses. Therefore, signaling mediated through the GK domain is a key for triggering calcineurin-dependent dispersal of GABA_A_Rs. The GK domain is known to interact with two proteins, GKAP^9^ and Axin^38^. S-SCAM-Axin interaction is more interesting because it connects S-SCAM to the GSK3β signaling pathway^39^ that was known to modulate GABAergic synapses^40^.

While we favor the model that S-SCAM overexpression causes the loss of existing GABAergic synapses, there remains a possibility that S-SCAM overexpression may also inhibit the formation of new GABAergic synapses. Time-lapse live-imaging of GABAergic synapses in S-SCAM over-expressing neurons would help to dissect out the relative contribution of the removal of existing synapses and the prevention of new synapse formation.

Previous studies showed that PSD-95 overexpression induces mis-localization of endogenous NL2, which in turn leads to the loss of NL2 from GABAergic synapses^41^ and reduces inhibitory synapse numbers^7^. However, we did not find evidence suggesting that S-SCAM overexpression causes similar mis-localization of NL2 to excitatory synapses at dendritic spines (Fig. 2g). Further studies are necessary to delineate the precise mechanism and signaling pathway by which S-SCAM overexpression induces GABAergic synapse loss.

S-SCAM can form dimers via inter-molecular interaction mediated by PDZ4 and PDZ5 domains^42^. Therefore, one caveat of our approaches is that the synaptic localization of S-SCAM mutants might be not rigorously tested. Nonetheless, our approaches effectively showed the functional differences in these mutants, presumably via a dominant-negative effect.

In addition to understanding the basic function of S-SCAM in GABAergic synapse formation and maintenance, our studies also have implication for the diseases associated with mutations in *S-SCAM* genes. Various mutations of the *S-SCAM* gene including copy number variations (CNVs) and single nucleotide polymorphisms were found in individuals with schizophrenia^14,43,44^. We previously showed that transgenic mice with increased levels of S-SCAM proteins have major abnormalities in glutamatergic synaptic function^13^. However, the hyper-glutamatergic model of schizophrenia is perceived unconventional since most of other genetic animal models of schizophrenia exhibits GABAergic deficits^45–47^. Our studies provide a new evidence supporting the pathogenic connection of hyper-glutamatergic function to GABAergic deficits in principal neurons.

Infantile spasms are associated with the loss of one copy of *S-SCAM* gene (haploinsufficiency). Numerous developmental syndromes are associated with partial chromosome deletions and insufficient protein amount is thought to be one of the main causes of haploinsufficiency^48,49^. Considering the preferential targeting of S-SCAM to excitatory synapses, it is conceivable that reduced S-SCAM levels may have more pronounced impact on GABAergic synapses. Our knockdown studies demonstrated that reduced S-SCAM protein levels indeed lead to the severe loss of GABAergic synapses, causing a shift in the E/I balance. Therefore, infantile spasms are likely conditions associated with insufficient S-SCAM protein levels in neurons.

In conclusion, our studies uncovered a profound role of S-SCAM in the proper formation/maintenance of GABAergic synapses and provide a clue to the pathogenic mechanisms associated with copy number variations of the *S-SCAM* gene in schizophrenia and infantile spasms.

## Methods

### Cultured rat hippocampal neurons

All experimental procedures involving the animals were approved by the Institutional Animal Care and Use Committee in the Medical College of Wisconsin and were carried out in accordance with the relevant guidelines and regulations. Dissociated rat hippocampal neuron culture was prepared from E18 embryos of Sprague Dawley rats as described^50^, plated on poly-D-lysine and laminin-coated coverslips in 12-well plates, and maintained in Neurobasal medium supplemented with B27 and Pen/Strep (ThermoFisher Scientific). Excitatory neurons (pyramidal neurons) were defined by their morphology (the presence of dendritic spines and greater than 4 major dendrites attached to the soma) and the absence of vGAT immuo-staining in the soma.

### Transfection and drug treatments

Hippocampal neurons were transfected at div 14 using Lipofectamine 2000 as described. For APV, CNQX, or FK506 treatment, drugs were added to the neuron culture 20 h post-transfection. COS7 cells were grown in DMEM supplemented with 10% FBS and gentamycin and transfected using Lipofectamine following the manufacturer’s suggested protocol.

### Immunocytochemistry and antibodies used

Transfected neurons were fixed at 3 days post-transfection. Immunocytochemistry was performed as described previously^11,51^. First, hippocampal neurons were fixed in 2% formaldehyde/1 × PBS/4% sucrose for 10 min followed by incubation in cold methanol (−20 °C) for 10 min. Fixed neurons were incubated with primary antibodies diluted in 1 × GDB (0.1% gelatin, 0.3% Triton X-100, 0.45 M NaCl, 17.7 mM sodium phosphate buffer, pH 7.4) in a humidified container overnight at 4 °C. Antibodies used and their dilution factors are: anti-GABA_A_R γ2 (1:250; Synaptic Systems), anti-GABA_A_R α2 (1:250; Synaptic Systems), anti-vGAT (1:800; Synaptic Systems), anti-NL2 (1:500; Synaptic Systems), anti-gephyrin (1:1000; Synaptic Systems), anti-β-Gal (Promega), and anti-myc (1:100; Santa Cruz Biotechnology).

Alexa 488, Cy3- or Cy5 conjugated secondary antibodies were used to visualize bound primary antibodies.

### Western blotting analyses

Total cell lysates were prepared by adding pre-heated 2 × SDS sample buffer (65 °C) to the wells of culture dishes after washing once in ice-cold 1 × PBS, separated on SDS-polyacrylamide gels, and transferred onto PVDF membrane. The membrane was blocked in 6% nonfat-dried milk/1× TBS-T. Primary antibodies, rabbit anti-GABA_A_R γ2 (1:250; Synaptic Systems), rabbit anti-pS237 GABA_A_R γ2 (1:250; PhosphoSoultions), mouse anti-actin (1:1000; Sigma), or rabbit anti-GFP (1:3000; Abcam), were diluted in the blocking buffer and incubated overnight at 4 °C. After washing in 1× TBS-T, membranes were further incubated for 1 h with HRP-conjugated secondary antibodies (GE Healthcare). Bound antibodies were detected by using SuperSignal West Pico Plus Chemiluminescent substrates (Thermo Scientific) and images were acquired by using luminescent image analyzer (ImageQuant LAS4000, GE Healthcare).

### Immunocytochemical image acquisition and analyses

Images were acquired by using a Nikon C1 plus laser scanning confocal microscope and 60 × objective (NA1.4). Acquired images (z-series stacks) were first converted to projection images (with maximal projection option) for analyses. Both image acquisition and analyses were done in a blind-manner. To measure puncta numbers and intensities per given neurons, five dendritic segments (~15–30 μm in length each) were selected from transfected neurons and their average values were used. After applying threshold, only puncta with more than 3 pixel sizes were counted. Their pixel area and total and average intensity were measured using SynPAnal software^52^. Colocalization is defined by puncta showing at least 1 overlapping pixel of two channels. For the quantification of surface GluA intensities, integrated intensity values (the sum of pixel intensity values for each pixel in a puncta) of sGluA immunofluorescence divided by the length of dendrites in dendritic segments were used to obtain average intensities in transfected neurons, and then normalized to the average values of those from neighboring non-transfected neurons. All data were transferred to GraphPad Prizm software for computation and graphical representation.

### Statistical analyses

All experimental data was collected at least from triplicate experiments using independent batches of neuron cultures. All data values represent means ± s.e.m. For multiple group comparisons, one-way or two-way ANOVA with Tukey’s multiple comparison post hoc test were used using GraphPad Prizm software. An unpaired t test was first used to determine the statistical significance for two groups. If there were significant differences in the variances of the two groups from an F test, Welch’s t test (assuming unequal variances) was used. *p* < 0.05 was considered significant. Box plots represent min and max (whiskers), a median and 25^th^ to 75^th^ percentile (box) of the data.

## Supplementary information

Supplementary Information.

## Data Availability

All materials, data, and associated protocols will be promptly made available to readers without undue qualifications in material transfer agreements.
