# Supplementary material for: Aberrant expression of S-SCAM causes the loss of GABAergic synapses in hippocampal neurons
Source: Sci Rep. 2020 Jan 9;10:83. doi: 10.1038/s41598-019-57053-y (PMC6952429; doi:10.1038/s41598-019-57053-y)
Supplement: Supplementary file 1 — Supplementary Information. [file 41598_2019_57053_MOESM1_ESM.pdf]

## Supplementary Information

# Aberrant expression of S-SCAM causes the loss of GABAergic synapses in hippocampal neurons

Seung Min Shin, Samantha Skaar, Eric Danielson, and Sang H. Lee

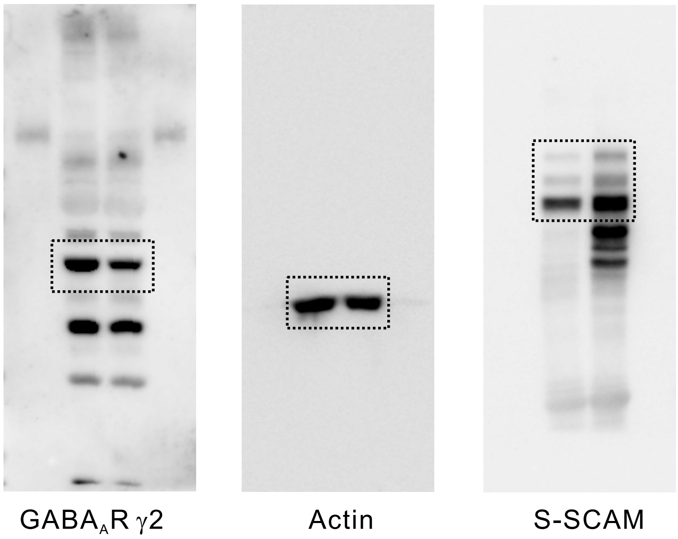


**Supplementary Figure 1**. Uncropped full size images of blots shown in Fig. 7c.
